# Efficacy of laparoscopic liver resection in colorectal liver metastases and the influence of preoperative chemotherapy

**DOI:** 10.1186/1477-7819-12-351

**Published:** 2014-11-21

**Authors:** Yoshihisa Kubota, Yuichiro Otsuka, Masaru Tsuchiya, Toshio Katagiri, Jun Ishii, Tetsuya Maeda, Akira Tamura, Hironori Kaneko

**Affiliations:** Division of General and Gastroenterological Surgery, Department of Surgery (Omori), Toho University School of Medicine, 6-11-1 Omori-nisi, Ota-ku, Tokyo, 143-8541 Japan

**Keywords:** Laparoscopic liver resection, Colorectal liver metastases, Preoperative chemotherapy

## Abstract

**Background:**

Since 1993, we have performed minimally invasive laparoscopic liver resection (LLR) to treat malignant liver cancer, including colorectal liver metastases (CLM). However, further studies are needed to accumulate sufficient evidence on the oncological outcome of LLR for CLM.

**Methods:**

To elucidate the efficacy of LLR for CLM, this study comparatively analyzed the invasiveness and short-term prognosis of LLR (n = 43 cases) and open liver resection (OR) (n = 62 cases) performed for CLM after 2006 and also investigated the safety of LLR following chemotherapy.

**Results:**

Compared with the OR group, the LLR group had significantly less blood loss (*P* < 0.001) and a shorter hospital stay (*P* < 0.001). The E-PASS scoring system was used to compare surgical invasiveness, and although the preoperative risk score did not differ between the groups, the surgical stress score and comprehensive risk score were significantly lower in the LLR group (*P* < 0.001). Concerning the survival rate and disease-free survival rate, there were no significant differences between procedures. However, more clinical cases and longer follow-up periods are needed to reach a definitive conclusion.

Preoperative hemanalysis, intraoperative bleeding, complications, and postoperative length of stay did not differ significantly between LLR patients with preoperative chemotherapy and those with surgery alone, indicating no adverse effects of chemotherapy.

**Conclusions:**

LLR can be an effective minimally invasive surgery in CLM patients receiving both perioperative chemotherapy and surgery. Because LLR is comparable with OR with regard to short-term oncological outcome, LLR may be a valuable option for CLM.

## Background

With the improvement in laparoscopic techniques and advances in surgical equipment, the safety and minimally invasiveness of laparoscopic liver resection (LLR) have been gaining recognition, and the procedure is on the verge of becoming a standardized surgical procedure due to the steadily increasing number of cases. However, no prospective randomized controlled study has been performed to compare oncological outcome between open liver resection (OR) and LLR in cases of malignant tumor. In 1993, several institutions including ours conducted studies on laparoscopic partial hepatectomy of hepatocellular carcinoma [1]. We later performed laparoscopic lateral hepatectomy for malignant tumors including colorectal liver metastasis (CLM), and in 1996, we reported the safety of LLR for CLM and its potential usage as a standard surgical technique [2]. We perform LLR in compliance with the indications, and to date, we have accumulated 211 cases of LLR, including 164 malignant cases. CLM is different from hepatocellular carcinoma in that the liver is otherwise normal and individual lesions are generally curable after resection with a free surgical margin, such as by partial hepatectomy. On the other hand, the disadvantages include abdominal adhesion due to surgery for the primary tumor, and more recently, hepatic damage caused by preoperative chemotherapy [3, 4]. However, no study has investigated the effect of preoperative chemotherapy on LLR. To elucidate the efficacy of LLR for CLM, we therefore compared the invasiveness and short-term prognosis of LLR and OR, and we evaluated the safety of LLR and the effect of chemotherapy on LLR.

## Methods

### Subjects

The Institutional Review Board of the Toho University Omori Medical Center approved this retrospective study (25 to 240). Our institution incorporated modified FOLFOX6 (mFOLFOX6) therapy in 2006 and molecularly targeted drugs, such as bevacizumab and cetuximab, in 2008. Between January 2006 and January 2013, 115 patients with CLM underwent hepatectomy, and of them, 105 were traceable and enrolled in this study. Clinical parameters were age, sex, primary tumor location, lymph node metastasis, time of liver metastasis, size and number of tumors, preoperative chemotherapy, intraoperative bleeding, operative duration, postoperative complications, and postoperative length of stay. Postoperative complications were evaluated in accordance with the Clavien-Dindo Classification [5], and the Estimation of Physiologic Ability and Surgical Stress (E-PASS) scoring system [6] was used to assess the low invasiveness of the surgical procedures. In addition, the rates of overall survival (OS) and disease-free survival (DFS) were compared.

To reveal the effects of preoperative chemotherapy on hepatectomy in the LLR group, the levels of transaminase and total bilirubin, prothrombin activity, platelet count, and the retention rate of indocyanine green 15 minutes after administration (ICGR15) were measured immediately before hepatectomy. Biopsy histopathology was performed according to the study by Rubbia-Brandt *et al*. [4] to elucidate hepatic damage due to chemotherapy. Clinical parameters were analyzed using the *t*-test and the X2 test, and OS and DFS were determined by Kaplan-Meier survival analysis followed by the log rank test. Statistical significance was set at *P* < 0.05.

### Principles of treatment for colorectal liver metastases

If a decision was made to resect metachronous liver metastases, the procedure was performed immediately by hepatectomy without preoperative chemotherapy.

With regard to simultaneous liver metastasis, six cycles of mFOLFOX6 + cetuximab or bevacizumab were given prior to hepatectomy in the following categories of patients: those >75 years old and those requiring >60% resection, portal vein embolization, and emergency surgery for conditions such as intestinal obstruction and perforation. Regardless of metachronous or simultaneous liver metastases, patients with an unresectable tumor received mFOLFOX6 + cetuximab as the first-line treatment and underwent hepatectomy as soon as the tumor became resectable. A recurrent liver tumor in the remnant liver was regarded as the first liver metastasis and treated by hepatectomy if resectable.

### Indications for laparoscopic liver resection and surgical options for colorectal liver metastases

The basic principles behind the indications and treatment for LLR entail the selection of cases in which LLR can be performed without compromising the safety and curability of patients, as in OR. Briefly, patients were subjected to general anesthesia according to the same protocol [7]. Preoperative liver function was evaluated collectively based on hemanalysis results, ICGR15, and remnant liver volume by computed tomography. If curable radical resection was applicable, there was no limit to the number of tumors. Although tumor size was not a limiting factor, it was often difficult to treat tumors larger than 8 cm. Partial resection was the fundamental surgical procedure, but systematic hepatectomy was sometimes selected depending on tumor location. In LLR, we aimed to resect with a 10-mm margin as long as the remnant liver remained functional; however, with regard to multiple tumors or tumors adjacent to the vascular system, the top priority was to ensure complete resection. In addition, many of the patients with CLM had undergone previous surgery for colorectal cancer, colostomy, or hepatectomy for recurrent tumor in the remnant liver. In these patients, a preoperative abdominal ultrasound was useful for ‘assessing the risk of intra-abdominal adhesions [8, 9]. For the assessment, a linear-type ultrasonic probe was used to evaluate differences in movement on the longitudinal side of the abdominal viscera accompanying spontaneous respiration, and the first trocar was inserted into the abdominal site where no adhesion was observed.

### Surgical techniques

We have previously reported detailed LLR techniques [10]. Patients with a tumor in the right lobe underwent surgery in the left semilateral position, and those with a tumor in the left lobe underwent surgery in the supine position. Although the trocar sites differed according to tumor location, an endoscopic trocar was inserted at the umbilicus, while surgical manipulation trocars were inserted into 2 to 4 ports. Exploration of tumor extent and its relationship with vascular anatomy and with other tumors in the liver were performed by intraoperative ultrasonography. Laparoscopic coagulation shears (Ethicon Endo Surgery Inc, Tokyo, Japan), a microwave tissue coagulator, and a monopolar sealer are generally useful for transection of superficial liver parenchyma. However, deeper transactions require careful use of ultrasonic dissection or the clamp crushing method. In this study, small vessels were transected by a bipolar sealing system or by clipping. Glisson’s capsule and hepatic veins were transected using a stapling device.

Pure laparoscopic liver resection has become the major procedure in systematic hepatectomy cases in recent years. However, depending on tumor size and location, other laparoscopy-assisted techniques can be used. Hand-assisted laparoscopic surgery is particularly useful for treating lesions in superior segments, such as segments VII and VIII.

## Results

### Laparoscopic liver resection versus open liver resection

One hundred-five patients with CLM who underwent hepatectomy at the Toho University Omori Medical Center were divided into the LLR (n = 43 cases, 41.0%) and OR (n = 62 cases, 59.0%) groups. There were no significant differences in age, sex, primary tumor location, or lymph node metastasis between the groups. In addition, the largest tumor diameter did not differ between the groups (*P* = 0.094); however, the LLR group had a significantly higher number of solitary tumors (*P* = 0.008). Preoperative chemotherapy was performed in 14 patients (32.6%) in the LLR group and in 29 patients (46.8%) in the OR group (*P* = 0.104). The LLR group had a significantly higher number of partial hepatectomy and lateral hepatectomy cases (*P* < 0.001). Intraoperative bleeding was significantly low in the LLR group (*P* < 0.001), with only one case of intraoperative transfusion (*P* < 0.01). Although the LLR group had no cases of R1 resection, there were four cases of R1 resection in the OR group. The prevalence of postoperative complications did not differ between the groups (*P* = 0.137). One patient in the LLR group had a grade I prolonged fever, while the OR group had one case of grade I intestinal obstruction, one case of grade III biliary fistula, one case of grade III intra-abdominal abscess, and three cases of grade II anastomotic leakage after simultaneous colectomy. The postoperative length of stay was significantly shorter in the LLR group (*P* < 0.001) (Table 1).

**Table 1:** **Demographic and clinical data of the study patients**

Parameter	LLR group (n = 43)	OR group (n = 62)	*P*value
**Age**	64.4 ± 11.4	65.5 ± 11.5	0.309
**Sex male/female**	22/21	40/22	0.122
**Primary tumor**			
Colon/rectum	24/19	40/22	0.243
Node-positive	24 (55.8%)	44 (70.1%)	0.083
**Liver metastasis**			
Metachronous/Synchronous	30/13	18/41	<0.001
Size of largest tumor			
<5 cm	35	42	
≥5 cm	8	20	0.094
Number of lesions			
1	27 (62.8%)	23 (37.1%)	0.008
2 to 4	15	27	
≥5	1	12	
**Perioperative chemotherapy**	14 (32.6%)	29 (46.8%)	0.104
**Type of resection**			
Partial resection	25	30	
Left lateral sectionectomy	11	7	
Segmentectomy, sectionectomy^a^	4	12	
Hemihepatectomy	3	11	
Extended hemihepatectomy	0	2	
Par, lat:seg, hm^b^	36:7	37:25	<0.001
**Operative time** **(min)**	333.9 ± 150.3	305.9 ± 107.1	0.149
**Operative blood loss** **(cc)**	287.3 ± 459.3	579.3 ± 392.0	<0.001
**Perioperative blood transfusion**	1 (2.4%)	13 (21.0%)	0.004
**R1 resection**	0 (0.0%)	4 (6.5%)	0.117
**Postoperative hospital stay** **(days)**	7.3 ± 1.8	14.2 ± 7.0	<0.001
**Complications** **(%)**	1 (2.4%)	6 (9.7%)	0.137
Clavien classification [5]			
I	1	1	
II	0	3	
III	0	2	
IV	0	0	
V	0	0	

^a^Except left lateral sectionectomy; ^b^Partial resection, left lateral sectionectomy: Segmentectomy, sectionectomy (except left lateral sectionectomy), hemi-hepatectomy.LLR, laparoscopic liver resection; OR, open liver resection.

**Figure 1:**
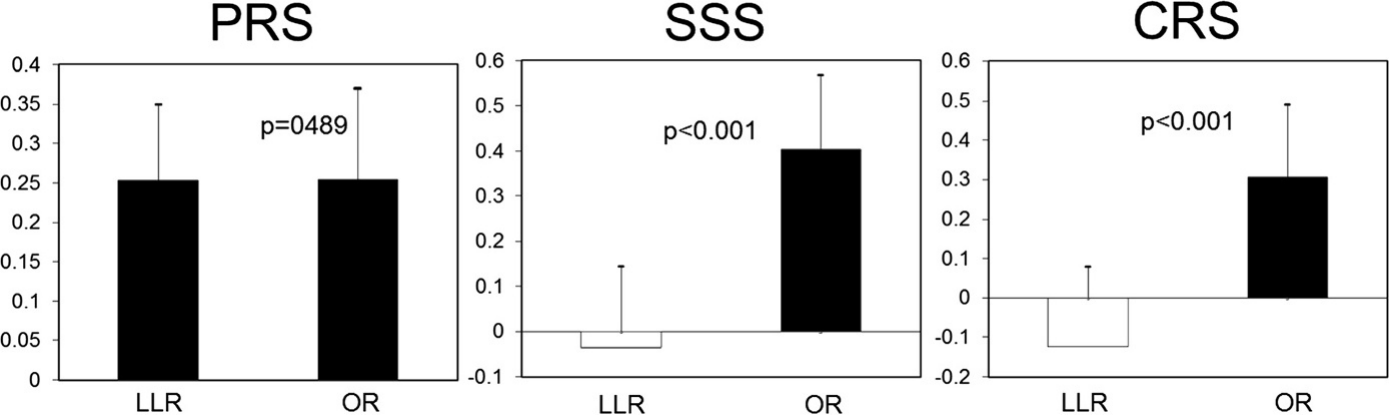
**Estimation of physiologic ability and surgical stress.** Preoperative risk score (PRS): -0.0686 + 0.00345X1 + 0.323X2 + 0.205X3 + 0.153X4 + 0.148X5 + 0.0666X6; X1, age; X2, presence (1) or absence (0) of severe heart disease; X3, presence (1) or absence (0) of severe pulmonary disease; X4, presence (1) or absence (0) of diabetes mellitus; X5, performance status index (0 to 4); X6, American Society of Anesthesiologists physiologic status classification (1 to 5). LLR, laparoscopic liver resection; OR, open liver resection. Surgical stress score (SSS): -0.342 + 0.0139X1 + 0.092X2 + 0.352X3; X1, blood loss/body weight (g/kg); X2, operation time (h); X3, extent of skin incision ((0) minor incision for laparoscopic or thoracoscopic surgery including scope-assisted surgery; (1) laparotomy or thoracotomy alone; (2) both laparotomy and thoracotomy). LLR, laparoscopic liver resection; OR, open liver resection. Comprehensive risk score (CRS): -0.328 + 0.936(PRS) +0.976(SSS). LLR, laparoscopic liver resection; OR, open liver resection.

**Figure 2:**
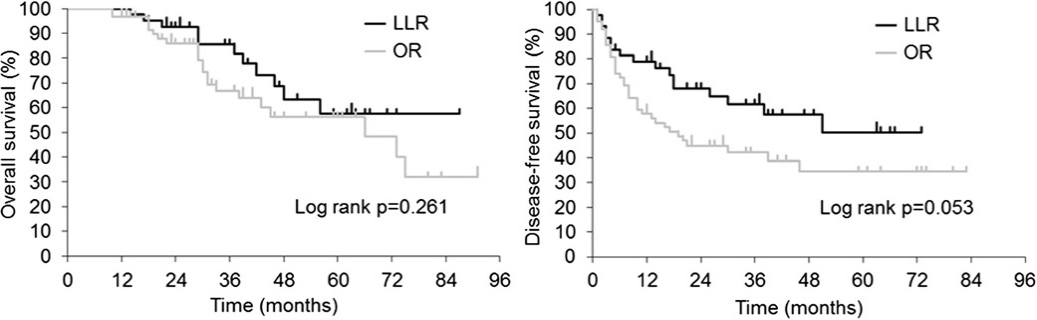
**Overall survival and disease-free survival in the study groups.** LLR, laparoscopic liver resection; OR, open liver resection.

**Figure 3:**
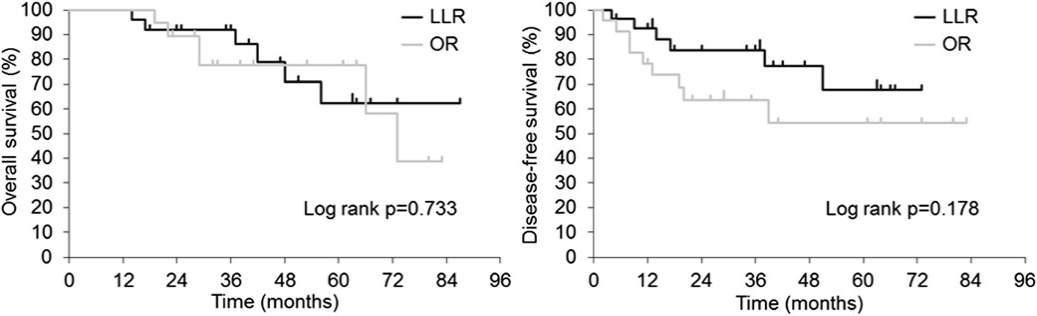
**Overall survival and disease-free survival in the single tumor cases.** LLR, laparoscopic liver resection; OR, open liver resection.

**Figure 4:**
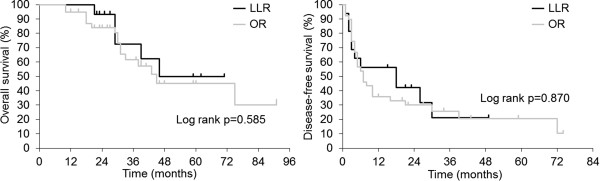
**Overall survival and disease-free survival in the multiple tumor cases.** LLR, laparoscopic liver resection; OR, open liver resection.

The E-PASS examination revealed a significantly lower surgical stress score (SSS) and comprehensive risk score (CRS), but not a preoperative risk score (PRS) (Figure 1) in the LLR group (*P* < 0.001). The median observation period was 36.8 months. The 3-year survival rate was 88.4% and 74.2% in the LLR and OR group, respectively, with no significant differences in OS (*P* = 0.261) or DFS (*P* = 0.053) (Figure 2). Tumor recurrence in remnant liver occurred in 16 cases (37.2%) in the LLR group and in 36 cases (58.1%) in the OR group. OS and DFS in the single tumor cases did not differ between the groups (*P* = 0.733 and *P* = 0.178, respectively) (Figure 3). In addition, although the number of cases is small, OS and DFS in the multiple tumor (two or more) cases have shown no difference between the groups (*P* = 0.585 and *P* = 0.870, respectively) (Figure 4).

### Safety evaluation of laparoscopic liver resection and the effect of preoperative chemotherapy

Thirty-two patients (76.1%) had a history of laparotomy by midline abdominal incision, four patients (12.5%) had undergone colostomy, and three patients (9.4%) had undergone more than two laparotomies. In these patients, preoperative abdominal ultrasound was performed to reveal intra-abdominal adhesions and contributed to the safe placement of the first trocar and successful completion of LLR. In the LLR group, preoperative chemotherapy included eight cases of mFOLFOX6 + bevacizumab, four cases of mFOLFOX6, one case of FOLFOX4 + bevacizumab and one case of FOLFIRI + bevacizumab with a median chemotherapy cycle number of 7.2 ± 2.4. Among the 43 cases of laparoscopic liver resection, there were 26 cases (60.5%) of pure laparoscopic liver resection, nine cases (20.9%) of hand-assisted laparoscopic surgery, and eight cases (18.6%) of laparoscopy-assisted liver resection. Repeat hepatectomy for recurrence in the remnant liver was performed in six patients (37.5%), of whom three underwent LLR (Table 2). The preoperative levels of transaminase and total-bilirubin, prothrombin activity, platelet count, and ICGR15 were normal, with no significant differences between the preoperative chemotherapy and surgery only groups. With regard to histological liver damage, five patients in the preoperative chemotherapy group had Rubbia-Brandt Classification grade 1 to 2 sinusoidal obstructive syndrome, with no significant differences in intraoperative bleeding, postoperative length of stay, or postoperative complications (Table 3).

**Table 2:** **Characteristics of patients undergoing laparoscopic liver resection**

**History of laparotomy by midline incision**	32/43	(76.1%)
Colostomy	4/32	(12.5%)
More than two laparotomies	3/32	(9.4%)
**Preoperative chemotherapy** **(n = 14)**		
mFOLFOX6 + bevacizumab	8	
mFOLFOX6	4	
FOLFOX4 + bevacizumab	1	
FOLFIRI + bevacizumab	1	
Median cycle number (course)	7.2 ± 2.4	
**Indications for preoperative chemotherapy**		
Received chemotherapy in other institution	7	
Presence of extrahepatic metastasis	3	
Large and multiple tumors	2	
Emergency surgery for primary tumor	2	
**Surgical procedures**		
Pure laparoscopic liver resection	26	(60.5%)
HALS	9	(20.9%)
Laparoscopy-assisted liver resection	8	(18.6%)
**Recurrence in the remnant liver**	16/43	(37.2%)
**Repeat hepatectomy**	6/16	(37.5%)
**Repeat hepatectomy underwent LLH**	3/6	(50.0%)

HALS, hand assist laparoscopic surgery;

LLH, laparoscopic liver resection; mFOLFOX6, modified FOLFOX6.

**Table 3:** **Characteristics of laparoscopic liver resection patients with preoperative chemotherapy and those with surgery alone**

Parameter	Preoperative chemotherapy (n = 14)	Surgery alone (n = 29)	*P*value
**AST** **(U/l)**	22.9 ± 6.6	23.8 ± 12.8	0.381
**ALT** **(U/l)**	19.4 ± 8.9	22.5 ± 15.2	0.207
**T-Bil** **(mg/dl)**	0.56 ± 0.13	0.67 ± 0.27	0.084
**PT** **(%)**	103.8 ± 10.1	105.6 ± 16.1	0.331
**Plt** **(10** ^**4**^ **/μl)**	18.7 ± 5.9	20.4 ± 6.2	0.205
**ICGR15** **(%)**	8.2 ± 4.1	8.0 ± 3.8	0.424
>10%	4 (28.6%)	4 (13.8%)	0.224
**Operative blood loss** **(cc)**	452.1 ± 692.4	207.7 ± 271.3	0.111
**Postoperative hospital stay** **(days)**	7.1 ± 1.1	7.4 ± 2.1	0.225
**Complications**	0	1	0.674
**Rubbia-Brandt Classification** **[** [7]**]**			
Grade 1	3		
Grade 2	2		
Grade 3	0		

ALT, aspartate aminotransferase; AST, alanine aminotransferase; ICGR15: indocyanine green dye retention rate at 15 min; Plt, Platelet; PT, prothrombin consumption test; T-Bil, total bilirubin.

## Discussion

In general, laparoscopy is considered less invasive than laparotomy; however, the methods of assessment vary widely. The E-PASS scoring system is often used for the assessment of surgical invasiveness because it collectively evaluates preoperative physiological function, intraoperative bleeding, operative duration, and incision size. It also expresses the risk of morbidity and mortality numerically. Although PRS did not differ between the LLR and OR groups, SSS and CRS were significantly lower in the LLR group. In addition, compared with the OR group, the LLR group had no serious complications, and the length of hospital stay was shorter, indicating the minimal invasiveness.

Hepatectomy is currently the first treatment option for CLM, and with a 5-year survival rate of 30 to 60%, it is considered the only curable option [11–13]. In Japan, partial hepatectomy has been the conventional surgical option for liver metastasis because there are no differences in the survival or recurrence rates between partial and systematic resection [14]. Thus, choice of surgery is not a prognostic factor [15]. In addition, the outcome is considered satisfactory if no tumor cells are exposed at the resection site in the remnant liver [16, 17], and partial hepatectomy is reportedly applicable for R1 resection in patients treated in preoperative chemotherapy [18]. Taking these factors into consideration, LLR appears to be a practical surgical procedure. This is also because partial hepatectomy is the standard surgical procedure in LLR and is considered the most appropriate surgical option for the inferior segments of the liver, such as segments IVa, V, and VI [11]. Although no difference in OS or DFS was observed between the LLR and OR group in this study, the rate of partial hepatectomy in single tumor cases, namely, a fair prognostic factor [19], was relatively high in the LLR group. However, the OS and DFS in single tumor cases did not significantly differ between the LLR and OR groups. The 5-year survival rate of LLR has been reported to be 50% for CLM [20]. Although no prospective randomized controlled study of LLR or OR performed for malignant tumors has been reported, retrospective studies have shown similar long-term prognosis in patients treated with LLR and OR for CLM [21, 22].

The efficacy of preoperative chemotherapy in patients with CLM has been recognized in recent years, and this modality has become the standard treatment in the United States and Europe [23, 24]. Recently, Scilletta *et al*. reported that neoadjuvant chemotherapy for CLM is not a significant risk factor for surgical site infections or complications according to the Clavien Dindo classification [25]. However, no study has investigated the effect of perioperative chemotherapy on LLR, and there remains a concern about the adverse effect of perioperative chemotherapy-associated liver damage on LLR. In this study, the perioperative chemotherapy group had a drug-free period of >4 or >6 weeks after treatment with mFOLFOX6 alone or with combination of bevacizumab, respectively. Consequently, no adverse effects of chemotherapy on hepatic functional reserve were observed prior to surgery; yet, gross and histological signs of hepatic sinusoidal dilatation were observed in five patients. Because management of bleeding is key to the completion of LLR, various energy devices and laparoscopic equipment including a stapler are used to control bleeding during liver tissue transection [26]. Intraoperative bleeding is a concern in patients with sinusoidal dilatation; however, as in cirrhosis in hepatocellular carcinoma, bleeding can be controlled with appropriate devices [7]. Accordingly, there were no differences in blood loss, postoperative complications, or postoperative hospital stay between the surgery alone and perioperative chemotherapy group in this study, failing to show the adverse effect, if any, of chemotherapy on LLR. In addition, rehepatectomy is often needed to treat tumor recurrence in the remnant liver. Intra-abdominal adhesions were relatively mild in patients treated with LLR in the previous surgery, and even though LLR was contraindicated in three of the patients on the basis of tumor location, rehepatectomy by LLR was successfully performed in the other three patients.

## Conclusions

The present study demonstrates the minimal invasiveness of LLR for CLM and suggests that LLR is comparable with OR with regard to short-term prognosis. No adverse effects of preoperative chemotherapy on LLR were observed, and LLR appears to be a beneficial option for repeated liver resection for CLM. LLR is thus a well-accepted surgical option for CLM, and chemotherapy drugs can be used without adverse effects with the proper knowledge of drug properties. However, to maximize the efficacy of LLR, it is necessary to comply with the indications to ensure the curability and safety of patients, to perform detailed preoperative diagnostic imaging, and to use the correct surgical devices.

## Abbreviations

CLM: colorectal liver metastases
CRS: comprehensive risk score
DFS: disease-free survival
E-PASS: Estimation of Physiologic Ability and Surgical Stress scoring system
LLR: laparoscopic liver resection
OR: open liver resection
OS: overall survival
SSS: surgical stress score
PRS: preoperative risk score.
